# Effect of Shenfu Injection on Isolated Empty Beating Hearts from Miniature Pigs

**DOI:** 10.21470/1678-9741-2019-0264

**Published:** 2020

**Authors:** Shijie Yin, Zhiqiang Feng, Ansheng Mo, Yi Ding, Jun Wu

**Affiliations:** 1 Department of Anesthesiology, The First Affiliated Hospital of Guangxi Traditional Chinese Medical University, Nanning, Guangxi, People’s Republic of China.; 2 Department of Cardiothoracic Surgery, The First Affiliated Hospital of Guangxi Traditional Chinese Medical University, Nanning, Guangxi, People’s Republic of China.

**Keywords:** Shen-Fu, Drugs, Chinese Herbal, Myocytes, Cardiac, Tissue Donors, Myocardium, Heart Transplantation, Physiological Phenomena, Erythrocytes

## Abstract

**Objective:**

To investigate the effect of Shenfu (SF) injection on donor heart preservation.

**Methods:**

Twelve pigs were randomly divided into SF group (n=6) and control group (n=6). After eight hours of perfusion, the differences in hemoglobin, the expression of Bcl-2 and BAX, and changes in the myocardial ultrastructure were compared to illustrate the effects of SF injection in heart preservation.

**Results:**

The differences in free hemoglobin between the SF group and the control group were statistically significant (*P*=0.001), and there was significant interaction of groups with times (*P*=0.019), but the perfusion time may not be associated with the hemoglobin concentration (*P*=0.616). According to Western blotting analysis, the expression of Bcl-2 was higher in the SF group than in the control group, while the expression of BAX was not different between the two groups. As to ultrastructural changes, both groups exhibited mitochondrial swelling and myofilament lysis, but the degree of damage in the SF group was smaller.

**Conclusion:**

Our study suggests that the application of SF injection for heart preservation may protect against cardiomyocytes and erythrocytes apoptosis, and Bcl-2 protein may play a role in these physiological processes.

**Table:** 

Abbreviations, acronyms & symbols	
ANOVA	= Analysis of variance
MPTP	= Mitochondrial permeability transition pore
mRNA	= Messenger ribonucleic acid
RBCs	= Red blood cells
SF	= Shenfu
TBST	= Tris-buffered saline
TNF-α	= Tumor necrosis factor alpha

## INTRODUCTION

In order to optimize the effect of heart preservation, new approaches have been explored. For instance, continuous perfusion of harvested hearts can safely prolong the preservation period compared with cold static storage^[1-3]^. In cold static storage, ischemia-reperfusion injury is inevitable, and continuous perfusion of harvested hearts can provide a near-physiologic environment for the heart by continuously infusing it with a warm, oxygen-rich medium. It cannot only provide sufficient nutrients, but also ensure the effective removal of metabolic waste. Furthermore, it also can lighten the ischemia-reperfusion injury induced by low temperature and avoid hyperkalemia. However, challenges still exist. Immune cells are infused with erythrocytes, metabolic substrates, and other beneficial substances, thus promoting a pro-inflammatory cytokine milieu after exposure to the *ex vivo* circuit^[4,5]^. The inflammatory environment has an adverse effect on the myocardium, vascular intima, and erythrocytes. Furthermore, severe mechanical damage and inflammatory mediators attack erythrocytes, resulting in increased free hemoglobin in the blood, which causes free hydroxyl production and lipid peroxidation, then leading to tissue damage.

Some traditional Chinese medicines are used as additives for heart preservation and can alleviate myocardial ischemia-reperfusion injury to some extent^[6,7]^. With the function of increasing blood flow, Shenfu (SF) injection may enhance the tolerance of organs to hypoxia^[8]^. Previous studies suggested that SF injection can improve myocardial metabolism by mediating the expression of Bcl-2 protein^[9]^ and inhibit myocardial ischemia-reperfusion injury in diabetic rats^[8]^. In addition, SF injection is also identified to be involved in the immune dysfunction by modulating the expression of complements and cytokines levels^[10]^, and Chen et al.^[11]^ report that SF injection may play a protective role in ischemia-reperfusion injury of liver transplantation. SF injection mainly consists of *Panax ginseng* and *Aconitum carmichaeli. Panax ginseng* is the steamed root of *Panax ginseng* C. A. Meyer and belongs to the family Araliaceae with the characteristics of a medicinal preparation in some countries. The active ingredients of *Panax ginseng* are the ginsenosides with a dammarane skeleton, which belong to dammarane type and include ginsenosides Rb1, Rb2, Rc, Rd, Rg1, Re, Rf, Rg2, and Rg3^[12]^. Recently, ginsenosides are reported to protect myocardial ultrastructure and reduce Ca^2+^ overload^[13]^. *Aconitum carmichaeli* is associated with heart rate, rhythm, blood pressure, and hemodynamics, and can protect myocardial cells^[14]^. Higenamine is the potent alkaloid isolated from *Aconitum carmichaeli,* with inotropic and chronotropic effects in cardiovascular system. Taken together, we hypothesized that the use of SF injection in continuous perfusion would attenuate the inflammatory response, improve microcirculation, and protect against myocardial injury, and this study was conducted to confirm this hypothesis.

## METHODS

Our Institutional Animal Care approved this study, and all animals were disposed according to the “Guide for the Care and Use of Laboratory Animals”^[15]^. Twelve Guangxi Bama miniature pigs weighing 25-30 Kg were randomly divided into experimental group and control group.

### Animal Preparation

After the animals were anesthetized by intramuscular injection of diazepam (1 mg/Kg) and ketamine (30 mg/Kg), a 24G venous indwelling needle was used to establish venous access via marginal ear vein. All pigs were intubated and mechanically ventilated. The respiratory rate was controlled at 20 times per minute, the tidal volume was 10 ml/Kg, and the inspiratory/expiratory ratio was 1:2. 1% to 2% isoflurane were used to maintain the anesthesia state. To perform hemodynamic monitoring and support, the right carotid artery and vein were cannulated. Then a median sternotomy was conducted. A blood crossmatch procedure was performed between the donor and the recipient to confirm the accurate matching prior to transfusion in case of insufficient blood supply.

### Preparation of Donor Hearts

After the great vessels were separated from the pericardium, heparin (400 U/Kg) was infused intravenously. The intact pericardium was preserved. Next, the brachiocephalic artery was cut off and the perfusion tube was inserted. Then, the donor blood was harvested through the superior vena cava and the right superior pulmonary vein, which was filtrated through leukocyte-depleting filters. After being arrested by 30 mL/Kg University of Wisconsin solution (Organ Recovery Systems, Itasca, Illinois, United States of America), the initial part of descending aorta and the two branches of aortic arch were cut off, and then the heart was removed. The drainage tube was placed through the pulmonary vein and the mitral valve, and the heart was inverted to achieve better decompression.

### Preparation of Perfusion

After filtrated out the leukocyte, the harvested blood was put into the perfusion device, which was described as a small cardiopulmonary bypass^[16]^, and mixed with 5 mL/Kg hydroxyethyl starch (Nanjing Zhengda Tianqing Co., Ltd., China) to perform oxygenation and temperature change. The perfusate consisted of sodium potassium magnesium calcium and glucose injection (10 mL/Kg, Jiangsu Hengrui Pharmaceutical Co., Ltd., China) and leukocyte-depleted blood (600 to 800 mL) with a total of 1100 to 1300 mL, and hematocrit was controlled at 20 to 25%. The SF injections (China Resources Sanjiu [Ya'an] Pharmaceutical Co., Ltd., China) were additionally applied in the experimental group by pre-filling in membrane oxygenation (0.4 mL/Kg/h) and continuous infusion (0.2 mL/Kg/h). The blood in the perfusion circuit was oxygenated with 95% oxygen and 5% carbon dioxide with a hollow-fiber membrane oxygenator (Maquet, German). Blood perfusion began within 10 minutes after arrest and was maintained at 32 ºC to 35 ºC for eight hours. The hemoglobin concentration in perfusate was detected at *T*_0_, *T*_1_ (one hour after blood perfusion), *T*_2_ (two hours after blood perfusion), *T*_3_ (three hours after blood perfusion), *T*_4_ (four hours after blood perfusion), *T*_5_ (five hours after blood perfusion), *T*_6_ (six hours after blood perfusion), *T*_7_ (seven hours after blood perfusion), and *T*_8_ (eight hours after blood perfusion) during preservation with hemoglobin colorimetric assay kit (Shanghai Chaoyan Biological Technology Co., Ltd., China). The perfusion flow was adjusted to achieve 60-80 mL/min and the perfusion pressure was controlled at 40-60 mmHg.

### Western Blotting Analysis

After continuous infusion for eight hours, a small piece of the left ventricular myocardium was homogenized in a cold lysis buffer supplemented with protease inhibitors, and protein concentrations were evaluated using a bicinchoninic acid protein assay kit (Elabscience, Wuhan, China) with Tubulin as loading control. The samples of tissue lysis were heated to 95 °C for five minutes. Protein samples (40 µg) were separated using 10% sodium dodecyl sulfate-polyacrylamide gel electrophoresis, and blotted onto polyvinylidene fluoride membranes overnight at 4 °C. Membranes were incubated with the appropriate primary antibody including anti-Bcl-2, anti-BAX, and anti-Tubulin, all from Elabscience, overnight at 4 °C. After washing with tris-buffered saline (TBST) three times, five minutes for each time, the secondary anti-rabbit immunoglobulin G antibody was then added and incubated for 30 minutes. After washing with TBST three times, proper amount of electrochemical luminescent substrate was added and incubated with the membranes in the dark. The results were scanned and processed by the ImageJ software.

### Myocardial Ultrastructure

After preservation for eight hours and cardioplegic arrest (Organ Recovery Systems, Itasca, Illinois, United States of America), left ventricular free walls were taken from two donor hearts from the experimental group and the control group. The tissues were processed into blocks for transmission electron microscopy^[17,18]^, of which glutaraldehyde and osmium tetroxide were applied as fixatives and spur was used as resin. A transmission electron microscope was applied for the ultrastructural findings of biopsies.

### Statistical Analysis

Measurement data were described as mean ± standard deviation, and differences among time points in the group were compared using one-way repeated measures analysis of variance (ANOVA). All statistical analyses were two-tailed, and a *P*-value of < 0.05 was identified as statistical significance. The IBM SPSS Statistics 22.0 software was used to perform these analyses.

## RESULTS

Basic characteristics of the experimental animals are shown in Table 1. The physiological measurements of pigs did not differ between the two groups (*P*>0.05). The total perfusate, anastomotic time, perfusion pressure, and perfusion flow were also not different (*P*>0.05).

**Table 1:** Comparison between SF group and control group.

Variables	SF group	Control group	*P*-value
Weight (Kg)	27.92±1.23	28.35±1.03	0.522
Male (n/%)	3 (50%)	3 (50%)	1.000
Age (month)	9.80±0.75	9.70±0.81	0.721
Perfusion pressure (mmHg)	54.67±4.13	53.00±2.97	0.441
Perfusion flow (mL/min)	70.52±4.35	68.60±4.61	0.476
Total perfusate (mL)	1180±34.29	1176.33±64.33	0.904
Cardiopulmonary bypass establishment time (min)	64.33±1.21	63.83±2.32	0.649

Quantitative data was shown as mean ± standard error. SF=Shenfu

**Fig. 1:**
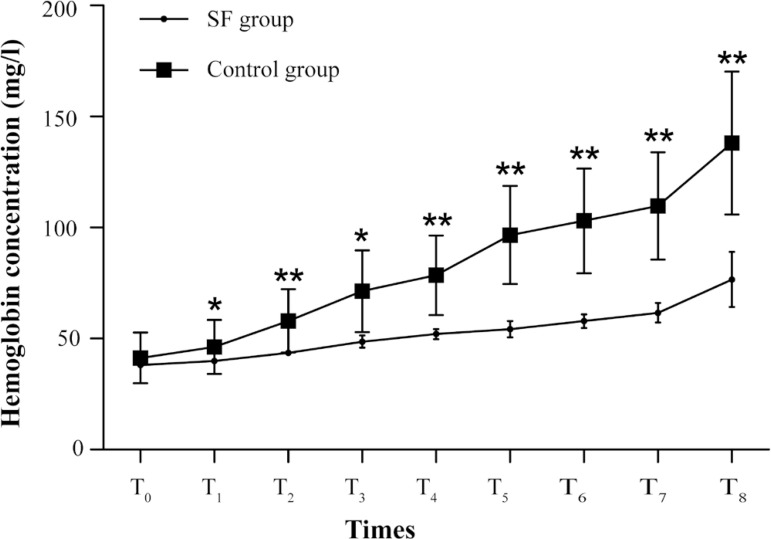
The increase of hemoglobin concentration in control group was significantly higher than in Shenfu (SF) group. The differences in the concentrations between the two groups were statistically significant (P=0.001), and there was significant interaction of groups with times (P=0.019), but the perfusion time may not be associated with the hemoglobin concentration (P=0.616). The concentrations of hemoglobin are shown as mean ± standard error. *P<0.05, **P<0.01.

In repeated measures ANOVA, Mauchly test of sphericity failed (*P*<0.001). So, the results of multivariate tests were adopted. According to these results, the differences in free hemoglobin between the SF group and the control group were statistically significant (*P*=0.001), and there was significant interaction of groups with times (*P*=0.019), but the perfusion time may not be associated with the hemoglobin concentration (*P*=0.616). The results are shown in Figure 1.

**Fig. 2:**
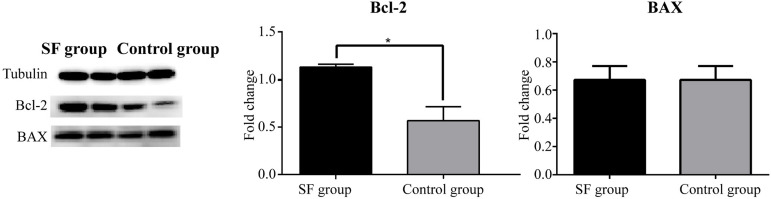
Protein expression of Bcl-2 and BAX in the Shenfu (SF) group and the control group. *P<0.05.

Western blotting analysis was conducted to semiquantify anti-apoptotic proteins, including Bcl-2 and BAX. The expression of Bcl-2 was higher in the SF group than in the control group, while the expression of BAX was not different between the two groups. Figure 2 displays the results of Western blotting analysis.

**Fig. 3:**
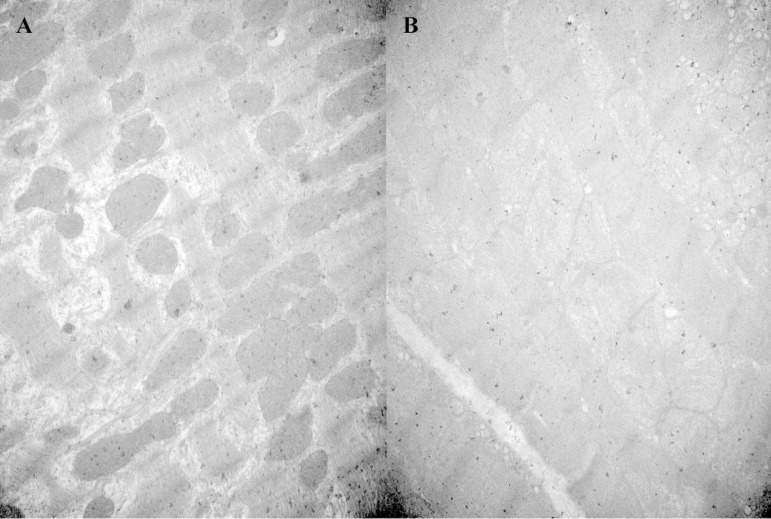
A) Shenfu (SF) group, B) control group. Both groups exhibited mitochondrial swelling and myofilament lysis, but the degree of damage in the SF group was smaller.

As to ultrastructural changes in Figure 3, both groups exhibited mitochondrial swelling and myofilament lysis, but the degree of damage in the SF group was smaller.

## DISCUSSION

Heart preservation in a normothermic beating state is considered as an ideal approach to prolong the preservation period as it can provide a near-physiologic environment for the heart, so this technology has been the focus over the past few decades. However, current studies on the approach cannot figure out myocardial ischemia. In the traditional Chinese medicine, SF injection has been routinely used for the treatment of cardiac diseases, such as acute myocardial dysfunction and chronic congestive heart failure^[19]^, and can protect against ischemia-reperfusion injury^[7,20,21]^, so we hypothesized that SF injection may alleviate the myocardial injury in heart preservation, and this study aimed to evaluate the effect of SF injection on the normothermic beating perfusion preservation of donor hearts. We did observe a protective effect in terms of hemoglobin, anti-apoptotic proteins, and ultrastructural changes.

During the perfusion process, anomalous mechanical and environmental factors influence the blood perfusion and cause damage to red blood cells (RBCs) through immediate and delayed hemolysis or the mechanical property changes of RBCs, which may impair microcirculation and tissue oxygen supply, and these processes would lead to the increase of hemoglobin concentration due to the release of RBCs^[22]^. Our study suggested that treatment with SF injection in perfusion can also improve microcirculation in perfusion, and possible reasons are as follows. Firstly, this protective effect may be involved in the role of SF injection in mediating inflammatory response, and SF injection can promote inflammatory response by lowering plasma concentration of tumor necrosis factor alpha (TNF-α) and can inhibit the overexpression of TNF-α messenger ribonucleic acid (mRNA) and interleukin-1 mRNA^[23]^. Secondly, SF injection has been reported to play a protective role in blood vessel spasm and microcirculation. Additionally, the components of SF, ginsenosides and aconitine, exert significantly protective effect on cardiovascular system by activating α and β receptors, thereby enhancing cardiac function as well as elevating and stabilizing blood pressure to relieve reperfusion injury^[24]^. However, inflammatory markers and related measurements were not investigated, and this was the limitation in our study; further studies on the abovementioned process should be focused on these. Based on the current observations and previous studies, SF injection may be used as an additive for heart preservation, which is regard as one of the commonly used drugs for the assistant treatment of cardiovascular diseases.

Bcl-2 family proteins participate in apoptosis in cardiomyocytes^[25,26]^, which is an important cellular process in myocardial ischemia-reperfusion injury. *In vitro* experiment demonstrated that SF injection has a protective effect on cardiac myocytes by mediating the expression of Bcl-2 protein^[10]^, especially in apoptosis induced by hypoxia/reoxygenation injury. As mentioned above, ischemia-reperfusion injury is inevitable in heart preservation, so SF may exert a protective effect against this injury in perfusion process. Furthermore, the addition of anti-Bcl-2 antibodies to the RBCs that had been loaded with Bcl-2 and Bcl-2/Δ34 can induce hemoglobin release from the RBCs under proper conditions, and this phenomenon suggested that a shortened fragment of Bcl-2, Bcl-2/Δ34, may have a functional activity^[27]^. BAX can promote the activation of apoptosis via multiple caspase-dependent pathways, and the collective effect of BAX and Bcl-2 would determine the initiation of mitochondrial apoptosis pathway. In our study, the expression of Bcl-2 was significantly increased in the SF group compared with the control group, and combined with the changes in concentration of hemoglobin during perfusion process, the interaction of SF injection with Bcl-2, not BAX, may play a role in cardiomyocytes and erythrocytes apoptosis.

Mitochondria are extremely important organelles, which accounts for 40-60% of the myocyte volume and can be associated with cardioprotection^[28]^. According to the changes in the myocardial ultrastructure, the SF group suggested milder damage than the control group. A previous study^[29]^ reported that the compound Chinese medicine “Kang Fu Ling” can protect against the myocardial injury by inhibiting the mitochondrial permeability transition pore (MPTP) opening that induced the early ischemia-reperfusion injury secondary to calcium overload, and the inhibitions of MPTP opening and calcium overloading are the critical processes in the postischemic treatment^[30]^. However, we can only identify that SF injection would have a protective effect against mitochondrial swelling in terms of the current findings, but the exact mechanisms are still uncertain, perhaps MPTP multiprotein complex is also involved in the protection of SF injection that influences the mitochondrial signal transduction^[31]^.

Perfusion management is a critical process in the normothermic beating preservation, of which the perfusion solution, perfusion flow, and perfusion pressure are the key elements^[32]^. In our study, the perfusion flow and pressure were indistinguishable between the two groups and were controlled at 40 to 60 mmHg and 60 to 80 mL/min, respectively. These data were determined previously^[33]^, and also were determined that the change of pressure from 100 to 50 mmHg would not influence oxygen uptake, coronary flow and cause ischemia^[34]^, and that a perfusion pressure of 40 to 60 mmHg can improve myocardial bleeding and edema^[35]^. Furthermore, lower perfusion flow would lead to hypoperfusion and deteriorate cardiac function. Therefore, our study controlled these factors to illustrate the effect of SF injection independently.

Several limitations also existed in our study. Firstly, the sample size of each group was limited, and the observations should be confirmed in other experimental studies. Another limitation was that how the SF injection protects against mitochondrial swelling should be investigated in further studies.

## CONCLUSION

Our study suggests that the application of SF injection for heart preservation may protect against cardiomyocytes and erythrocytes apoptosis, and Bcl-2 protein may play a role in these physiological processes.

**Table:** 

Authors' roles & responsibilities	
SY	Drafting the work; final approval of the version to be published
ZF	Revising it critically for important intellectual content; final approval of the version to be published
AM	Substantial contributions to the design of the work; final approval of the version to be published
YD	Substantial contributions to the acquisition and interpretation of data for the work; final approval of the version to be published
JW	Substantial contributions to the analysis of data for the work; final approval of the version to be published
